# The Application of Machine Learning: Controlling the Preparation of Environmental Materials and Carbon Neutrality

**DOI:** 10.3390/ijerph20031871

**Published:** 2023-01-19

**Authors:** Zhenxing Wang, Yunjun Yu, Kallol Roy, Cheng Gao, Lei Huang

**Affiliations:** 1 South China Institute of Environmental Sciences, Ministry of Ecology and Environment of the People’s Republic of China, Guangzhou 510655, China; 2 Institute of Computer Science, Faculty of Science and Technology, University of Tartu, 51009 Tartu, Estonia; 3 College of Hydrology and Water Resources, Hohai University, Nanjing 210098, China; 4 School of Environmental Science and Engineering, Guangzhou University, Guangzhou 510006, China

The greenhouse effect is a severe global problem. Various countries (both developed and developing) have formulated policies and developed new technologies to prevent carbon emissions. Rapid industrialization has prompted the European states of France, the United Kingdom, Germany, and Denmark to promulgate new laws to realize the goal of carbon neutrality in 2050 [1,2,3,4,5]. The other big industrial nations of the United States, Canada, Japan, and Australia also promise to achieve the target of carbon neutrality by 2050 [6,7,8]. As a largest developing country, China also announces its carbon neutrality target by 2060 [9,10,11]. The research and development of low-carbon technologies are critical in achieving the target, as is energy-efficient wastewater treatment.

Traditional sewage treatments are energy-hungry and also need various harmful chemical reagents [12]. Renewable energy from solar or hydrogen energy fits the choice for wastewater treatment. The designs of new solar cell materials, hydrogen production, and their efficient storage are of utmost importance, without which carbon neutrality is impossible. Organic matter should be adequately decomposed before being released into the natural water bodies. Free radicals generated during advanced oxidation can produce damaging effects [13,14]. Catalytic materials are instrumental in adsorbing heavy metals or negative ions [15,16]. Thus, the optimal choice of adsorption materials (e.g., perovskites) is necessary for catalyzing and adsorbing pollution in water and is reported by our previous work [17]. The other significant members in the list are as follows: membrane technology, ion exchange, and the electrochemistry method [18,19,20,21].

The green synthesis of reagents influences its performance. Besides green synthesis being a low-carbon technology, it also bypasses the environmentally unfriendly requirements of hydrothermal, ultrasonic, microwave, and high-temperature calcination, as well as coprecipitation and impregnation methods. Traditional synthesis runs many iterations to find an optimal synthetic route for a chemical compound. For example, temperatures of more than 900 °C are applied to calcine metal–organic frameworks [22,23]. The coprecipitation and sol–gel reaction method search various stoichiometric combinations (percentages) of ions to prepare composite material [24,25]. The different organic ligands are recursively iterated for discovering new covalent organic frameworks [26]. These experiments waste many chemical reagents and energy sources. Thus, the low-carbon synthetic route is a non-tractable problem because of the exponential complexity of generating paths. Machine learning and statistical methods are now mature enough to tame the complexity of chemical synthesis methods and to even suggest new methods.

Machine learning is gaining popularity and finds applications in Big Data, image recognition, biomedical imaging, text classification, and even in environmental governance, among other things [27,28,29,30,31,32]. The different contenders in machine learning are decision trees, deep neural networks, random forests, support vector machines, Bayesian networks, boosting, and bagging [33,34,35,36]. Guo et al. reported the adsorption and desorption of heavy metals on the interface of sediment by using the Bayes algorithm [37]. Chen et al. also applied the Bayesian algorithm to design nanofiltration membranes, which could facilitate the removal of contaminants in the waste water [38]. These (and many other researches) show immense promise of machine learning in designing and synthesizing materials for treating wastewater.

In recent years, many researchers reported various studies of material synthesis [39]. Ryan P. Adams and Abigail G. Doyle et al. found that Bayesian optimization was more effective than human decision making [40]. In chemical synthesis, James M. Tour et al. used the parameters of mass, capacitance, voltage, pre-treatment, and duration, and then applied an ensemble of models to forecast the yield of graphene, an extremely important material in wastewater treatment [41]. Furthermore, graphene is a dominating material in the treatment of waste water. Cory M. Simo and Janardhan Rao Doppa also reported the use of Bayesian optimization for designing nanoporous materials [42]. This has brought an impetus for the adsorbing materials or membrane materials for sewage treatment.

In conclusion, machine learning will be increasingly used in the design and development of the green synthesis of environmental materials that help to achieve carbon neutrality. It can provide the following directions in the design: (1) the design of solar cell materials with a higher conversion rate; (2) the design of higher producing and storing hydrogen rate in hydrogen energy; (3) the adsorption of materials with a higher adsorbing capacity and fast adsorption; (4) the catalytic materials with higher efficiency for CO_2_ conversion; (5) the membrane materials with a higher efficiency of retention rate; (6) more models with machine learning and deep learning. The development promotes material synthesis, water treatment, and carbon neutrality.

This Special Issue is built to promote the researchers who want to have an academic crossover by using artificial intelligence in environmental engineering. This provides us with more ideas and guidance on developing environmental engineering in the future. Thank you to everyone who wants to submit, has submitted, or can contribute to this Special Issue.

## Data Availability

The data presented in this study are available on request from the corresponding author. The data are not publicly available due to privacy.
